# Groundwater nitrate pollution risk assessment based on the potential impact of land use, nitrogen balance, and vulnerability

**DOI:** 10.1007/s11356-023-30850-9

**Published:** 2023-11-16

**Authors:** Robert Duda, Robert Zdechlik, Jarosław Kania

**Affiliations:** Faculty of Geology, Geophysics and Environmental Protection, Department of Hydrogeology and Engineering Geology, AGH University of Science and Technology, Al. Mickiewicza 30, 30-059 Kraków, Poland

**Keywords:** Nitrogen surplus, Nitrate, Fertilization, Manure, Adverse impact, Pollution

## Abstract

The predicting groundwater nitrate pollution risk, especially in terms of changes in fertilizing, has not been fully investigated so far. In particular, there is no comprehensive method to assess this risk in areas of different land use type, and not only in agricultural areas. The aim of this study was to develop a novel multicriteria methodology for groundwater nitrate pollution risk assessment, which meets these issues. A further aim was to determine how much this risk would change if the amount of organic and synthetic fertilization was reduced. An assumption was that groundwater pollution risk is a combination of the potential adverse impacts of land use, fertilization, and intrinsic groundwater vulnerability to pollution. The impact of fertilization was holistically evaluated by balancing nitrogen from spatially differentiated the size of the breeding, species of livestock, manure and synthetic fertilizers input, and spatially differentiated topsoil, with nitrogen uptake by different crops. The nitrate concentration in the leachate was used as a measure of the impact of fertilization. This concentration was compared to the natural baseline nitrate concentration in groundwater. Three fertilization scenarios for groundwater pollution risk assessment in two study areas were discussed. Under typical agricultural, climatic, soil, and geological conditions in Europe for the current total fertilization level of 95-120 kg N ha^−1^ groundwater nitrate pollution risk is low and moderate, but for fertilization of 150-180 kg N ha^−1^, a reduction in the total fertilization (synthetic and manure) by 40 to 50% may be required to achieve low risk of degradation of natural groundwater quality. Predictive simulations of groundwater nitrate pollution risk confirmed that reducing synthetic and organic fertilization has an effect, especially in areas with intensive fertilization. This method may allow for a holistic and scenario-based assessment of groundwater pollution risk and may help decision-makers introduce solutions to manage this risk under conditions of climate change, preservation of groundwater quality, and food security.

**Supplementary Information:**

The online version contains supplementary material available at 10.1007/s11356-023-30850-9.

## Introduction

Nitrogen fertilization poses a serious risk to natural groundwater quality in areas of concentrated agriculture and livestock husbandry, and rural areas (Potter et al. 2010; Bouwman et al. 2013; Wang et al. 2019; He et al. 2022; Alam et al. 2023). Overfertilization results in a nitrogen surplus, which is then transported in leachate to groundwater (Anas et al. 2020; Klages et al. 2020; De Vries et al. 2022; Su et al. 2022). In addition, gaseous nitrogen emissions, including from volatilization from fertilizers, increases greenhouse gas emissions (Dong et al. 2006; Chadwick et al. 2011; Anas et al. 2020).

Groundwater risk assessment is the prediction of how much groundwater quality is at risk if a certain anthropogenic pressure occurs. Predictions of groundwater risk can aid decision-makers and stakeholders in managing these risks to maintain good quality groundwater for the environment, terrestrial and aquatic ecosystems, and population supply. Groundwater nitrate pollution risk is a kind of a subgroup of groundwater risk. Major studies on groundwater nitrate pollution risk assessment have been briefly described by Xu et al. (2023). In addition, recently risk assessments have been performed among others by Teng et al. (2019), by Wu et al. (2020), and by Atoui and Agoubi (2022). Jahromi et al. (2021) conducted this assessment using a modified classical index method. Huan et al. (2020) have performed groundwater nitrate pollution risk assessments of the groundwater source field. At the regional scale, numerical modeling of nitrate mass transport has been performed by Zhao et al. (2022) and Xu et al. (2023). Zhang et al. (2022) adopted a quantitative approach considering pollution loading. Gao et al. (2014) modeled nitrogen loading by considering relevant elements of a soil nitrogen balance. Ortuzar-Iragorri et al. (2018) calculated the gaseous and leached nitrogen balance. Koh et al. (2021) estimated the nitrogen input from synthetic fertilizers by considering the nitrogen demand of crops. Kazakis et al. (2015) performed groundwater pollution risk assessments by considering the risks from areal and local sources of pollution, using intrinsic groundwater vulnerability assessment methods dedicated to different types of aquifers. Kazakis and Voudouris (2015) estimated nitrogen losses from the soil based on various data using indices produced by sophisticated modeling. An approach based on the nitrogen input hazard index was applied by Orellana-Macías and Perles Roselló (2022).

However, despite these investigations, the problem of groundwater nitrate pollution risk prediction is still open. In addition to the intrinsic vulnerability of groundwater and the impact of areal and local land use, spatially variable nitrogen inputs from organic and synthetic fertilization and the soil nitrogen balance should be considered to make reliable predictions. A fully holistic approach has not been used in the groundwater nitrate pollution risk investigations so far. This approach in the predicted impact of fertilization evaluation should include balancing nitrogen loads originated from spatially differentiated (i) breeding, (ii) species of livestock, (iii) manure and synthetic fertilizers input, and (iv) topsoil, with nitrogen uptake by different crops. Obtained this way, the nitrate concentration in the leachate provides a correct quantitative measure of the degree of the adverse effects of fertilization on the natural baseline nitrate concentration in an aquifer. Apart from that, natural groundwater quality is characterized by groundwater chemical constituent baseline concentrations (Edmunds and Shand 2008). The baseline nitrate concentrations (Shand and Edmunds 2008) are significantly lower than the threshold nitrate concentrations for groundwater bodies with good chemical status and water intended for drinking purposes—which are typically 50 mg L^−1^. Therefore, the subject of assessing the risk of groundwater pollution—one of the components of the natural environment—should be natural groundwater quality, instead of the legally recognized good chemical status. Another issue is that groundwater risk assessments are usually carried out for rivers catchments. In addition to fertilized land, there may be other forms of land use that pose a potential threat to the natural quality of groundwater. This means that the evaluation of the groundwater pollution risk in such a catchment should also take into account potential adverse impacts of non-agricultural land use types.

The groundwater nitrate pollution risk affects also habitats—mainly ecosystems reliant on surface expression of groundwater. This includes springs, streams and rivers (especially during low-flow conditions), and wetlands. These are biotic aquatic species as invertebrates, fish, and amphibians, whose health status and well-being depend on water quality (Dudgeon et al. 2006; Reid et al. 2019; Cantonati et al. 2020).

Therefore, the first goal of this study was to develop a novel holistic method that effectively combines the all aforementioned factors and conditions of groundwater nitrate pollution risk assessment. Another goal was to determine how much the groundwater nitrate pollution risk in arable areas would decrease if organic and synthetic fertilization was reduced, as specified in the adopted scenarios. To achieve these objectives, this study considers spatially variable (i) synthetic fertilization, (ii) livestock husbandry and manure production, (iii) soil type, (iv) cultivated crops, and (v) aquifer recharge. The consideration of soil type in the groundwater nitrate pollution risk assessment is needed because there are often made on a regional scale and as the study area increases; spatial variation in soil types tends to increase. Different soil types have different levels of naturally occurring soil nitrogen (Wang et al. 2009; Jia et al. 2017; Yao et al. 2019) which should be included in the overall nitrogen balance. Lack of consideration of the spatial variability of soil nitrogen in the spatial assessment of nitrogen input may result in inaccurate assessment of groundwater risk.

The novelty of our study is its proposal of a more comprehensive approach to the methodology of groundwater nitrate pollution risk assessment than is typically applied. Our novel method comprises a quantitative evaluation of the impact of fertilization and qualitative assessment non-agricultural land use impact—based on its own classification. Groundwater intrinsic vulnerability is assessed according to the new approach, also. The effect of nitrogen fertilization on the risk of groundwater contamination was assessed based on the nitrate concentration in the leachate. This concentration results from the balance of the input of nitrogen from manure, synthetic fertilizer, and soil nitrogen with the amount of nitrogen that cultivated crops can uptake in different soils. The risk forecast is scenario-based and assesses the effects of reduced fertilization, among other factors, resulting from reduced animal husbandry. Another novelty is the adoption of natural groundwater quality, namely, using nitrates’ natural hydrogeochemical baseline as a threshold value for assessing the adverse impact of fertilization, instead of using the nitrate threshold value for groundwater bodies with good-quality status. Our classification of potential adverse impacts of land use types in the catchment area is also new.

This study was carried out for two areas differing from geological settings and fertilization intensity. Duda et al. (2020) carried out groundwater pollution risk assessment based only on its vulnerability to pollution and simplified assessment of land use potential impact at one of the areas. In the second area, no local studies related to the threat and protection of groundwater have been carried out so far.

## Method

The applied groundwater nitrate pollution risk assessment method merges the assessments of (i) the potential adverse impact of land use; (ii) the impact of fertilization estimated by balancing the predicted manure, synthetic, and soil nitrogen input with the possible uptake by different crops cultivated on variable soils; and (iii) the groundwater intrinsic vulnerability to pollution.

### Potential impact of land use (LU)

The potential adverse impact of areal and local land use (*LU*_*i*_) was determined based on the classification (Table 1), with an adjustment for the effect of the number of types of land use with potential adverse impacts on the overall value of this factor in an individual calculation cell. This classification was based on the principle that an increase in the degree of adverse impacts of various land use types is affected by:

- an increase in the mass of chemicals processed, manufactured, or stored (which is related to the land use types and pollution locations) that can potentially be emitted from a given location into groundwater;
- an increase in the concentrations of chemical compounds in the leachate;
- an increase in the toxicity of chemical compounds emitted from a particular pollution location into groundwater;
- a decrease in depth from the level of a given pollution location to the groundwater table; and
- a decrease in the thickness of natural or artificial insulation of the pollution location from the ground or an absence of insulation.

**Table 1:** Classification of potential adverse impacts of land use types (*LU*_*i*_)

Areal land use type	Grade	Rating *LU*_*i*_
• Forests and bushes • Meadows	No impact	0
• Urbanized areas with sewer networks with negligible leakage • Extensive grazing areas • Orchards, plantations, and allotment gardens with extensive cultivation	Low	1
• Urbanized areas with sewer networks with limited leakage • Orchards and intensive cultivation plantations • Areas irrigated by treated municipal wastewater and gray water • Intensive grazing areas	Moderate	2
• Urbanized areas with sewer networks with significant leakage • Areas irrigated by untreated municipal wastewater	High	3
• Urbanized areas without sewage systems	Very high	4
Local land use facility		
• Non-isolated landfills of inert waste and waste rock	Low	1
• Surface tanks for liquid chemical compounds • Surface tanks for liquid fuels • Industrial dry cleaners • Rubber industry production facilities • Paper mills • Food processing facilities • Power plants/combined heat and power plants • Landfills and dumps of municipal waste, industrial waste, power plant waste, slag heaps isolated from the ground • Chemical mineral storage sites isolated from the ground • Open-pit mines of raw materials other than metal ores • Plant protection products storage facilities	Moderate	2
• Underground tanks for liquid chemical compounds • Underground tanks for liquid fuels • Fuel storage facilities • Pipelines with liquid chemical compounds • Metal ore processing and enrichment plants • Classification yards • Landfills and dumps of municipal waste, industrial waste, power plant waste, slag heaps poorly isolated from the ground • Chemical mineral storage sites poorly isolated from the ground • Cemeteries	High	3
• Landfills and dumps of municipal waste, industrial waste, power plant waste, slag heaps non-isolated from the ground • Metal ore tailing ponds • Chemical mineral storage sites non-isolated from the ground • Oil mines, oil transfer stations, oil pipelines • Oil refineries, chemical plants • Iron and steel mills • Large metallurgical industry plants • Electroplating facilities • Leather tanning facilities • Metal ore mines liquidated by flooding • Leaking manure and slurry tanks, uncovered slabs with organic fertilizers	Very high	4

The degree of the potential adverse impact of individual local land use types was adopted by considering their typical technical and technological characteristics. Therefore, in the case of facilities designed to significantly counteract groundwater pollution, the assessment of potential adverse impacts adopted in this classification may be overstated.

The factors shaping the potential groundwater risk at the regional scale are characterized by spatial variability; therefore, analysis and evaluation require the discretization of the area into calculation cells (raster cells). Calculations were performed using GIS. The resulting raster map contained the calculated risk values for individual cells. A raster size of 500 × 500 m was used. This size was a compromise between the precision of risk mapping and the legibility of the resulting map at a regional scale. A cell size that is too small limits the readability of spatial variation because of the difficulty in observing individual cells with different ranks.

When several land use types (*LU*_*i*_) were present in a given calculation cell, a total assessment of their potential impact was calculated in two stages. First, a weighted average of the ratings of the various land use types present within the cell was calculated, where the weight was the number of land use types with a given adverse impact. Next, to consider the effect of the number of all land use types on the overall value of the *LU* factor for a given calculation cell*,* the weighted average value was multiplied by the correction factor *f* (Eqs. 1, 2).

1 $$LU=f\cdot {LU}_{i}$$

2 $$f=\sqrt[4]{{n}^{3}}$$

where *n* is the number of land use types within the considered calculation cell.

If there was more than one land use type in the calculation cell, the adjusted value of this factor was directly proportional to the number and weight of each risk. The adopted (Eq. 2) degree of the root (fourth) and exponent of the power (third) are the lowest integer values that ensure the correctness of the risk assessment approach used. The overall value of the potential adverse impact of multiple land use types calculated for a cell will always be greater than the value of the largest rating of a single threat. Using an *LU* value calculated as a weighted average of distinct threats in an individual cell could result in an overall adverse impact rating, which is less than the rating of the largest impact occurring in the cell. However, using the maximum value from the ratings for different types as the overall rating for a given cell is also inappropriate because it does not consider the effect of an increase in the number of threats on increasing the overall adverse impact occurring within that cell. Adopting the maximum value would result in the following: regardless of the number of threats present in the cell, the assessment of potential adverse impacts would consider only this threat, ignoring the potential negative impacts of other threats.

### Adverse impact of fertilization (F_N_)

The adverse effects of nitrogen fertilization on arable land (*F*_*N*_) were quantitatively estimated in a manner similar to that applied by Duda et al. (2021). The magnitude of the impact was assumed to depend on the balance of nitrogen input relative to the crop nitrogen uptake capacity (*L*_*N*_). The balance considered the spatial distribution of (*i*) livestock husbandry intensity and manure generation, (*ii*) mineral fertilization, (*iii*) topsoils, and (*iv*) cultivated crop species. This balance omitted nitrogen losses associated with possible surface runoff and volatilization. The most unfavorable model for the groundwater risk was therefore adopted. The balance (*L*_*N*_) was the difference between the total nitrogen input from natural (*N*_*an*_) and synthetic fertilization (*N*_*am*_) and soil nitrogen (*N*_*as*_), and the maximum amount that can be taken up by particular types of crops grown on a given topsoil type (*N*_*d*_):

3 $${L}_{N}={N}_{an}+{N}_{am}+{N}_{as}-{N}_{d}$$

4 $${N}_{as}={N}_{s}\cdot \mathrm{\alpha }$$

where *N*_*s*_ is the soil mineral nitrogen reserve according to the soil agronomic category (Supplementary Table S1), and *α* is a coefficient approximating the efficiency of soil nitrogen on crop yield relative to the effect of synthetic nitrogen (Table S2).

The amount of nitrogen in livestock manures depends on various factors and can be estimated using various methods (Šebek et al. 2014; Velthof et al. 2015). We calculated it according to the following formula:

5 $$N_{an}={\sum }_{i=1}^{n}A_{i}\cdot N_{Fm_{i}}\cdot \beta_{i}$$

where* i* is the number of animal species; *A* is the number of animals of a given species; *N*_*Fm*_ is the approximate rate of nitrogen contained in manure (kg N head^−1^ year^−1^) (Table S3); and *β* is a coefficient determining the approximate effectiveness of manure nitrogen on crop yield relative to synthetic nitrogen (Table S2).

The input of active nitrogen from synthetic fertilizers was determined based on statistical data of the amount of fertilizer used in the year and the nitrogen content per unit weight of fertilizer product.

Plants have a natural capacity to take up nitrogen, which depends on various factors and varies among species (Glass 2003; Masclaux-Daubresse et al. 2010; Milroy et al. 2019). The amount of nitrogen that could be taken up by the crops (*N*_*d*_) was calculated according to Table S4. The weighted average amount of nitrogen for each crop in the rotation system on the soil of the medium agronomic class was considered, where the weight was the area occupied by each species.

The adverse effects of fertilization (*F*_*N*_) were determined as the nitrate concentration in the leachate recharging the aquifer:

6 $${C}_{NO3}=({L}_{N}/RCH)\cdot 443$$

where *L*_*N*_ is the result of nitrogen fertilization balance (kg N ha^−1^ year^−1^), *RCH* is the aquifer recharge (mm year^−1^), and 443 is the unit conversion.

Nitrate baseline concentration in the groundwater usually does not exceed 9 mg NO_3 _L^−1^ (Shand and Edmunds 2008; Huang et al. 2013). An assumption was that a lack of deterioration in natural groundwater quality implied no impact of fertilization. The classification and rating of the adverse impact of fertilization (*F*_*N*_) based on nitrate concentration in the leachate were adopted (Table 2).

**Table 2:** Classification and rating of the adverse impact of fertilization (*F*_*N*_) based on nitrate concentration in the leachate

Nitrate concentration (*C*_*NO3*_) (mg L^−1^)	Impact	Rating
≤ 10	No	0
10 − 20	Low	1
20 − 50	Moderate	2
50 − 100	High	3
> 100	Very high	4

### Intrinsic vulnerability (V)

Groundwater vulnerability to pollution was assessed using the DIRECT index. This method is a modified DRASTIC method (Aller et al. 1987). Therefore, two original factors regarding the natural properties of the aquifer, aquifer lithology and hydraulic conductivity, were omitted from the assessment. These factors do not affect the time, velocity, and contaminant load transported downward from the terrain surface to groundwater through the vadose zone (Mishima et al. 2011; Jiménez-Madrid et al. 2013; Orellana-Macías and Perles Roselló 2022). The other five original factors were considered to have an actual influence on groundwater’s intrinsic vulnerability to pollution: depth to the groundwater table (*D*), impact of the lithology of the vadose zone (*I*), net recharge (*REC*), topsoil type (*T*), and terrain topography (Supplementary Figs. S1 and S2). The weights and scores associated with these factors were assumed to be the same as those in the original DRASTIC scale. Four classes and ratings of groundwater intrinsic vulnerability (*V*) were adopted, depending on the DIRECT index: ≤ 95 low vulnerability (rating 1), > 95–110 moderate (2), > 110–125 high (3), and > 125 very high (4).

### Groundwater nitrate pollution risk (R)

The groundwater nitrate pollution risk grade in the calculation cell considered depends on the evaluation of the total rating obtained using the following equation:

7 $$R=LU+{F}_{N}+V$$

where *LU* is the potential adverse impact of land use type on groundwater quality (according to Table 1) calculated by Eq. (1), *F*_*N*_ is the adverse impact of fertilization assessed based on nitrogen balance, and *V* is the rank of groundwater vulnerability. The applied classification of groundwater nitrate pollution risk had five grades depending on the *R* rating (Table 3).

**Table 3:** Classification of the groundwater nitrate pollution risk (*R*)

Risk	Rating
Very low	≤ 2
Low	2 − 4
Moderate	4 − 6
High	6 − 8
Very high	*R* > 8

## Material

### Study areas

**Fig. 1:**
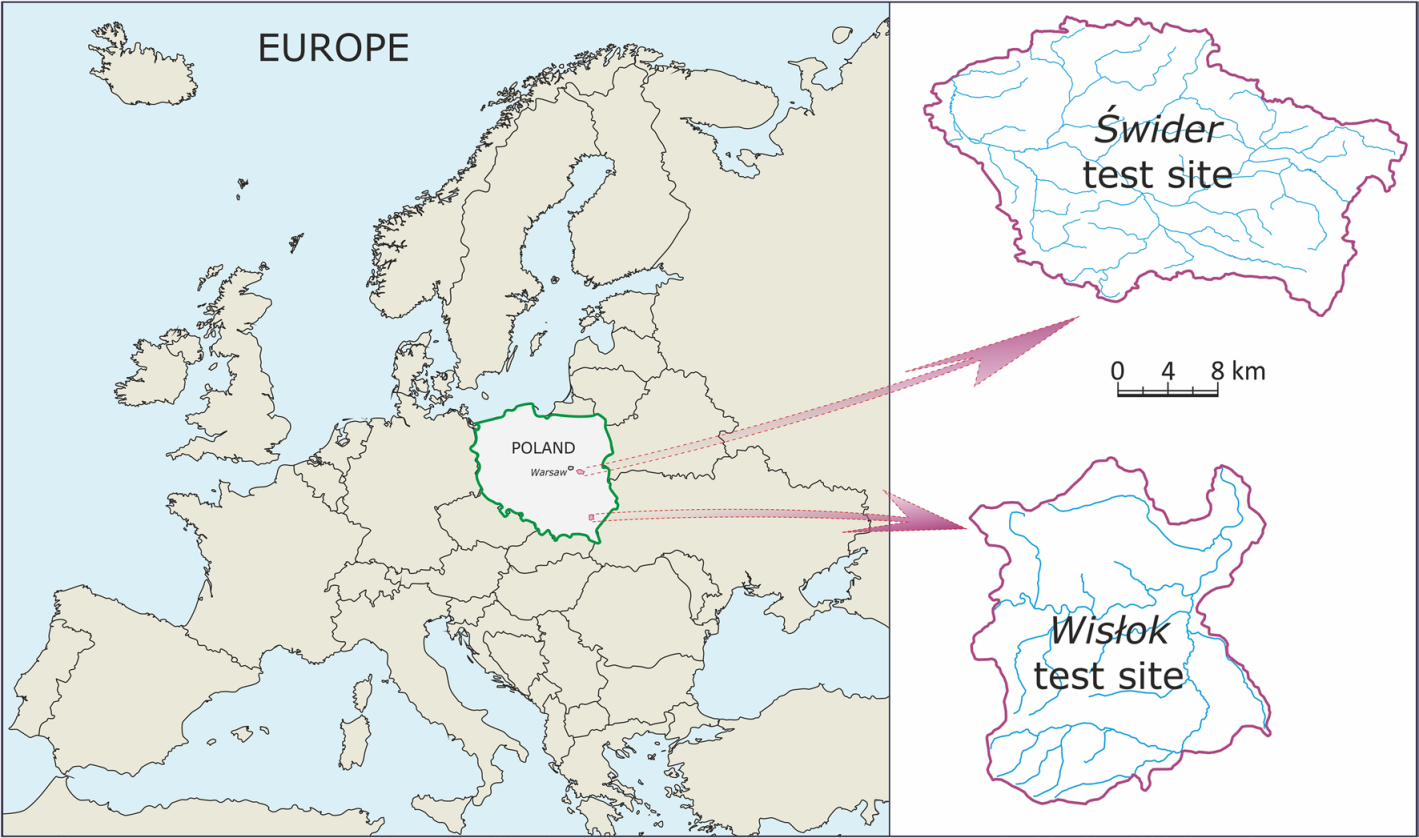
Location of test sites

This study was performed at two test sites located in Poland, Europe (Fig. 1). The reason for the investigation in the two areas was that they differ in geomorphology, thickness and lithology of the vadose zone, crop diversity, size of livestock farming, and the level of fertilization on arable land (Table S5). The study of two areas allowed for analyzing the sensitivity and effectivity of the method used to change the values of parameters affecting groundwater pollution risk.

**Fig. 2:**
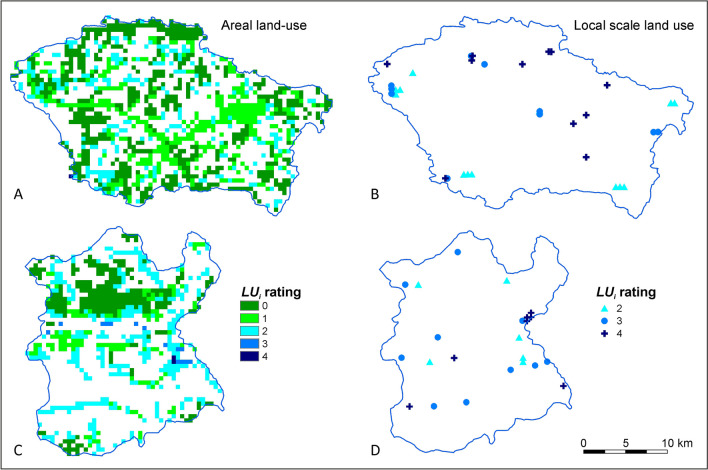
Spatial distribution of land use potential adverse impact in Świder (**A**, **B**) and Wisłok (**C**, **D**) test sites. *LU*_*i*_ rating according to Table 1

The Świder River catchment area (552 km^2^) is located in the lowland region with a temperate climate. Elevations of the terrain in the study area are in the range of 100–210 m a.s.l. There is an unconfined porous groundwater body and the depth to the groundwater table usually does not exceed 7 m*.* The characteristics of the vadose zone lithology and the depth of the groundwater table were obtained from geological and hydrogeological maps at a scale of 1:50,000 from the Polish Geological Institute, Warsaw, Poland (www.pgi.gov.pl/en). Annual precipitation was 560–623 mm. Precipitation data were obtained from the Institute of Meteorology and Water Management, Warsaw, Poland (www.imgw.pl). Forests covered 24% of the area, pastures and orchards with extensive cultivation 21%, various types of built-up and industrial areas in total 6%, and fertilized arable land 49% (Fig. 2A). The ranges of areal land use types were obtained from the CORINE Land Cover system developed by the European Environmental Agency (http://clc.gios.gov.pl). Potential local sources of groundwater pollution exist in this area (Fig. 2B).

The Wisłok River catchment area (442 km^2^) is located in the upland and foothill regions. There are greater terrain differences, and the elevations are in the range of 160–400 m a.s.l. As a result, with the exception of river valleys, the thickness of the vadose zone often exceeds 8 m. Weakly permeable loams and silts were observed in the vadose zone of the southern part of the area. The average annual rainfall was 690 mm. Forests covered 13% of the area, pastures and orchards 12%, various types of built-up and industrial areas in total 13%, and fertilized arable land 62% (Fig. 2C). Various local sources of groundwater pollution were identified (Fig. 2D).

At both test sites, in addition to cereals, potatoes, beets, rapeseed, and other vegetables were grown in varying proportions (Table S5). Synthetic and organic fertilizers (manure and slurry) were also used. Both areas raised mainly hens, chickens, cows, beef cattle, hogs, and turkeys in varying proportions (Table S5). Statistical data on agricultural crop species and areas, livestock species and populations, and synthetic nitrogen fertilizer inputs were obtained from a database on the Statistics Poland website (https://bdl.stat.gov.pl). The data collected in this database were assigned to administrative units rather than river catchment areas. For this reason, statistics were related to this study’s catchment areas using the proportion of the area of each administrative unit to the catchment area.

In the study area, soils with different soil nitrogen contents (very light, light, medium, and heavy) occurred in varying surface proportions (Table S5). The soil ranges of the different agronomic categories were adopted according to a 1:300,000 map prepared by the Institute of Soil Science and Plant Cultivation, Puławy, Poland (https://en.iung.pl). Owing to the use of crop rotation, directly relating crop species to the soil type in which they were grown was not possible. Thus, we assumed that all crop species were grown on a given soil type in an area proportion similar to that in each administrative unit.

### Research scenarios

To improve the scope of the sensitivity analysis of the method applied to changes in parameter values, we assumed three groundwater pollution risk scenarios as a result of fertilization. The adoption of fertilization-level scenarios also made it possible to assess the degree of change in the projected groundwater nitrate pollution risk as a result of reduced anthropogenic pressure due to the impact of environmental policies and economic conditions. The approach to fertilization and husbandry should change, as recommended by the European Green Deal (European Commission 2019) and the EU climate policy (European Parliament 2016). In addition, the new economy affects the fertilization rate because of an increase in the price of synthetic fertilizers. This increase is due to an increase in the price of natural gas, the primary raw material for nitrogen fertilizer production.

We assumed that in the medium-term future (until 2030), land use types and their extent in the test sites would not change significantly. Scenario I assumed land use, fertilizer levels, animal husbandry, and cultivated crop species as of 2020. Scenario II assumed a 25% reduction in synthetic nitrogen fertilization levels compared with 2020 at the Świder test site and a 10% reduction at the Wisłok test site. These assumptions are an example of the synergistic effect of Green Deal policies and a significant increase in natural gas prices. Scenario III assumed (i) synthetic fertilization as in scenario II; (ii) in the area with intensive husbandry (Świder test site), the level of organic fertilization was less than that in 2020 owing to reducing husbandry by 50% and/or allocating manure for biogas production; (iii) in the area with moderate husbandry (Wisłok test site), husbandry was unchanged. These assumptions are an example of the synergistic effects of the Green Deal, the implementation of policies to reduce atmospheric nitrogen emissions and climate change, and the effects of rising natural gas prices.

## Results and discussion

**Fig. 3:**
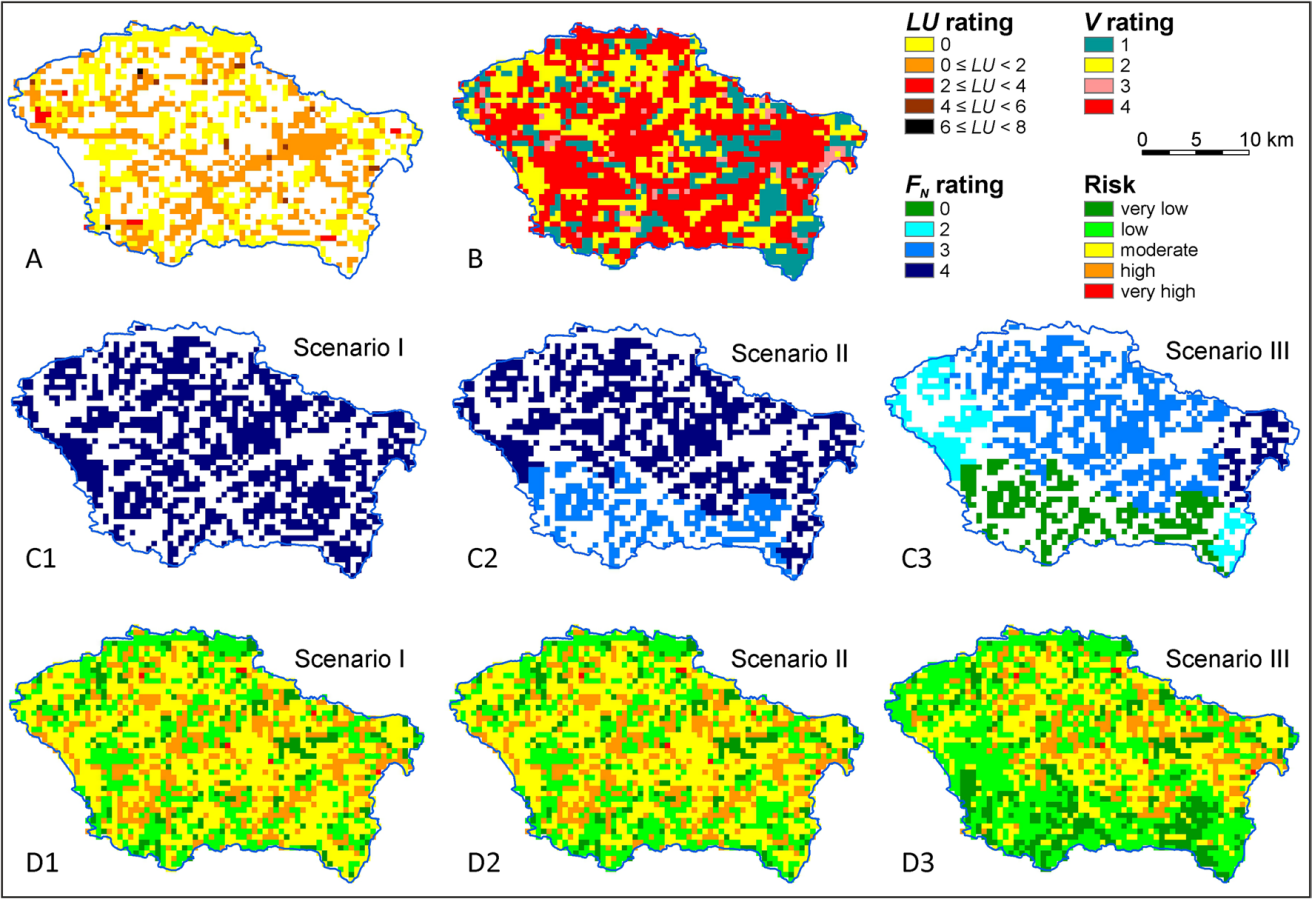
Groundwater nitrate pollution risk assessment of the Świder test site—areas with the land use impact assessment *LU* (**A**), groundwater vulnerability *V* (**B**), areas with quantitatively estimated fertilization impact *F*_*N*_ (C1, C2, C3) and groundwater risk *R* (D1, D2, D3)

The first stage of the groundwater nitrate pollution risk assessment determined the spatial distribution of the risk factor ratings using GIS. The spatial variability of potential adverse impact of land use types (*LU*) at both test sites is shown in Figs. 3A and 5A. The spatial variability of groundwater vulnerability (*V*) is shown in Figs. 3B and 5B. The results of the scenario calculations for the nitrogen load balance from fertilization, including the areas of crops and the amount of applied nitrogen inputs from organic and synthetic fertilizers, were summarized in worksheets (Table S5). Based on the nitrogen balance, the spatial variability of the potential adverse fertilizing impact *F*_*N*_ in the adopted scenarios was determined (Figs. 3C and 5C).

In both catchments, land use is similar and usually does not result in a high risk of groundwaters pollution in non-arable areas (Figs. 3A and 5A). Apart from fertilized land, differences in the degree of risk between catchments result from differences in the intrinsic vulnerability of groundwater, which is mainly related to the different thickness and soil lithology. In the Świder test site, almost half of the area is characterized by very high vulnerability, while in the Wisłok test site it does not exceed 5% of the area (Figs. 3B and 5B).

In the catchment area with intensive fertilization (Świder test site), the current total nitrogen input was 170–225 kg N ha^−1^, including 130–180 kg N ha^−1^ from fertilization, depending on the subarea (Table S5). A subarea is a fragment of a country’s administrative units within a catchment. Scenario 1 (current fertilization level) was dominated by zones with a moderate risk of groundwater pollution (45% of the area), and zones with high and low risk accounted for 20% and 25%, respectively (Figs. 3D1 and 4A). Areas with very low risk include zones where groundwater vulnerability was low and moderate and where there were forests whose impact was *LU* = 0 (Fig. 3D1 vs. A and B).

**Fig. 4:**
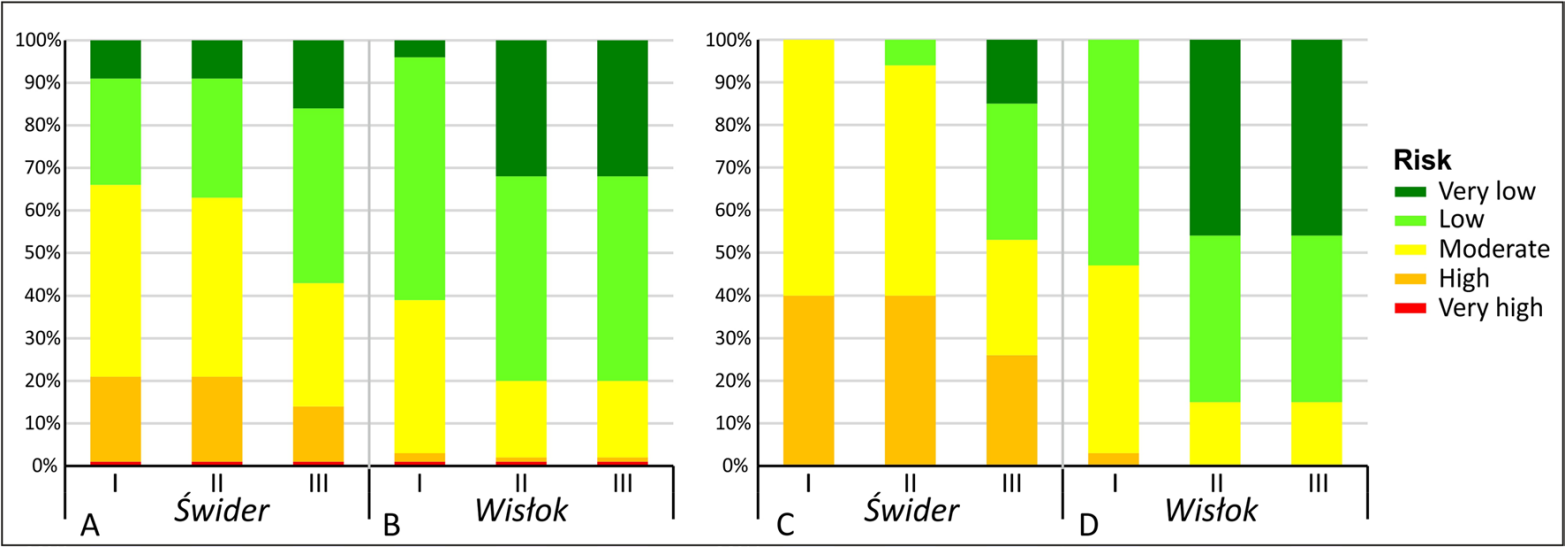
Proportion of areas with a given groundwater nitrate pollution risk in the Świder (**A**) and Wisłok (**B**) test sites and in fertilized areas in the Świder (**C**) and Wisłok (**D**) depending on the pressure scenario (I, II, III)

Reducing the level of synthetic fertilization by 25% (scenario II) did not significantly change the degree of adverse fertilization impact (*F*_*N*_) or, consequently, groundwater nitrate pollution risk (Fig. 3C2 vs. C1, and D2 vs. D1). The adoption of severe fertilizer restrictions in scenario III (in addition to a 50% reduction in husbandry) significantly reduced the impact of fertilization (Fig. 3C3). As a result, low-risk zones dominated, the area of very low-risk zones increased, and the moderate- and high-risk zones decreased (Figs. 3D3 and 4A). Zones of very high risk occurred sporadically and were associated with potentially adverse impacts of local land use types, whose locations, extent, and degrees of potentially adverse impacts did not change across scenarios.

**Fig. 5:**
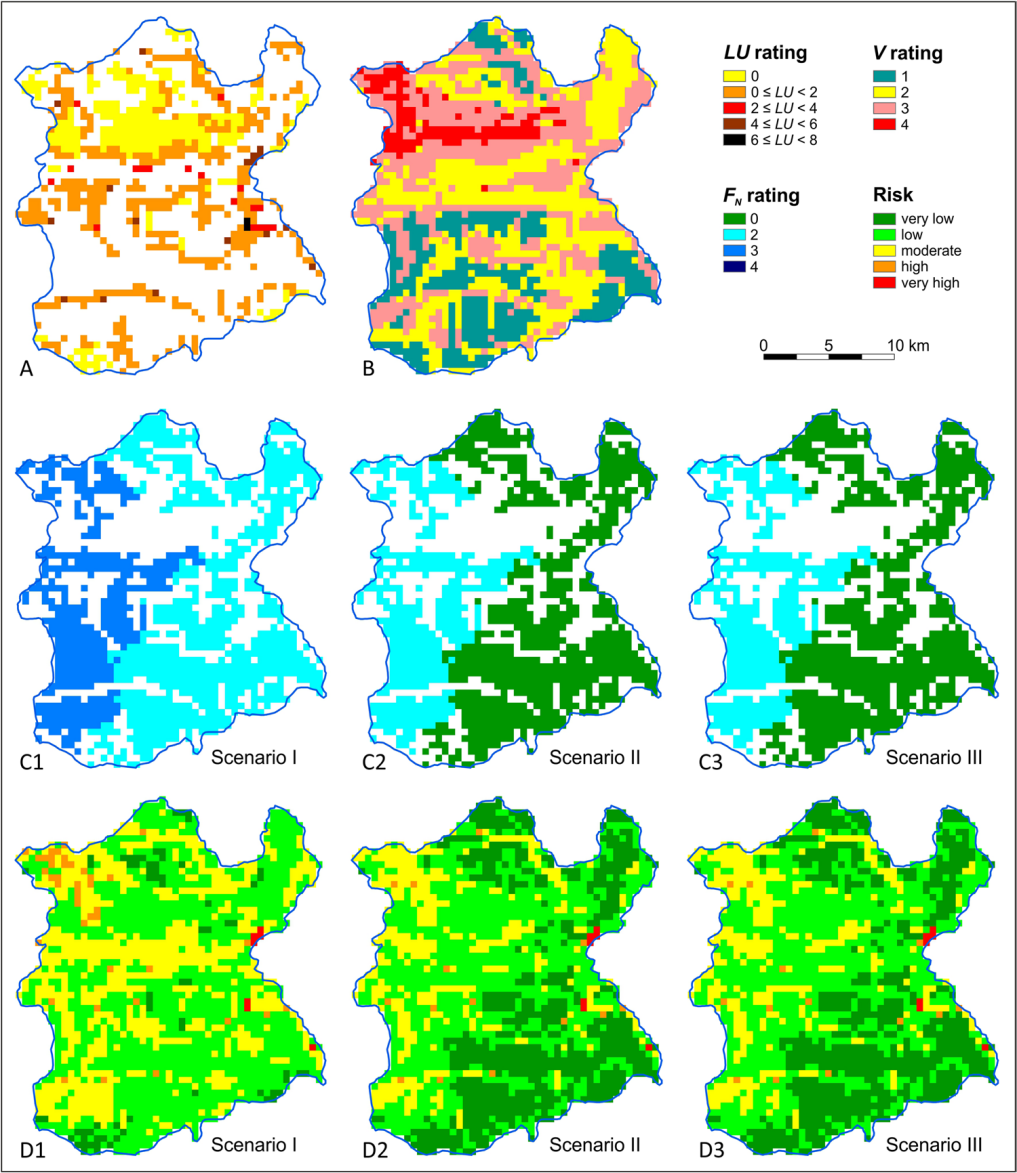
Groundwater nitrate pollution risk assessment of the Wisłok test site: areas with the land use impact assessment *LU* (**A**), groundwater vulnerability *V* (**B**), areas with quantitatively estimated fertilization impact *F*_*N*_ (C1, C2, C3), and groundwater risk *R* (D1, D2, D3)

In a catchment area with moderate fertilization and husbandry (Wisłok test site), similar patterns were observed, but on a smaller scale than that of the test site with intensive fertilization. This difference is due to the less productive nature of agriculture relative to a Świder catchment area. At the Wisłok test site, the current total nitrogen input was 135-165 kg N ha^−1^, including 95-120 kg N ha^−1^ from fertilization, depending on the subarea (Table S5). Therefore, it was lower than in the Świder catchment area by an average of 30%. Scenario I was dominated by zones with low and moderate groundwater nitrate pollution risk: 57% and 36% of the area, respectively (Figs. 4B and 5D1). Regions with high and very high risk occurred only occasionally because of local land use types. Zones with very low risk generally included areas with very low groundwater vulnerability (Fig. 5D1 vs. B).

The scenario II forecast showed a reduction in the degree of the impact of fertilized areas (*F*_*N*_) (Fig. 5C2 vs. C1). The spatial distribution of risk in much of the area showed a reduction in risk from low to very low and from moderate to low (Figs. 4B and 5D2 vs. D1). This result confirmed that greater reductions in synthetic fertilization than those initially adopted in the scenarios were unnecessary.

Considering only agricultural areas where fertilization occurs, the reduction in the groundwater nitrate pollution risk as a result of reduced fertilization, depending on the scenario, was more pronounced. The proportion of areas where the groundwater nitrate pollution risk was high or moderate decreased significantly. However, in the case of catchment with intensive fertilization, this decrease only occurred under scenario III (Fig. 4C). In the test site with low productive agriculture, the level of fertilization corresponding to scenario II caused a significant reduction in the groundwater nitrate pollution risk through the appearance of a large proportion of areas of very low risk (Fig. 4D).

**Fig. 6:**
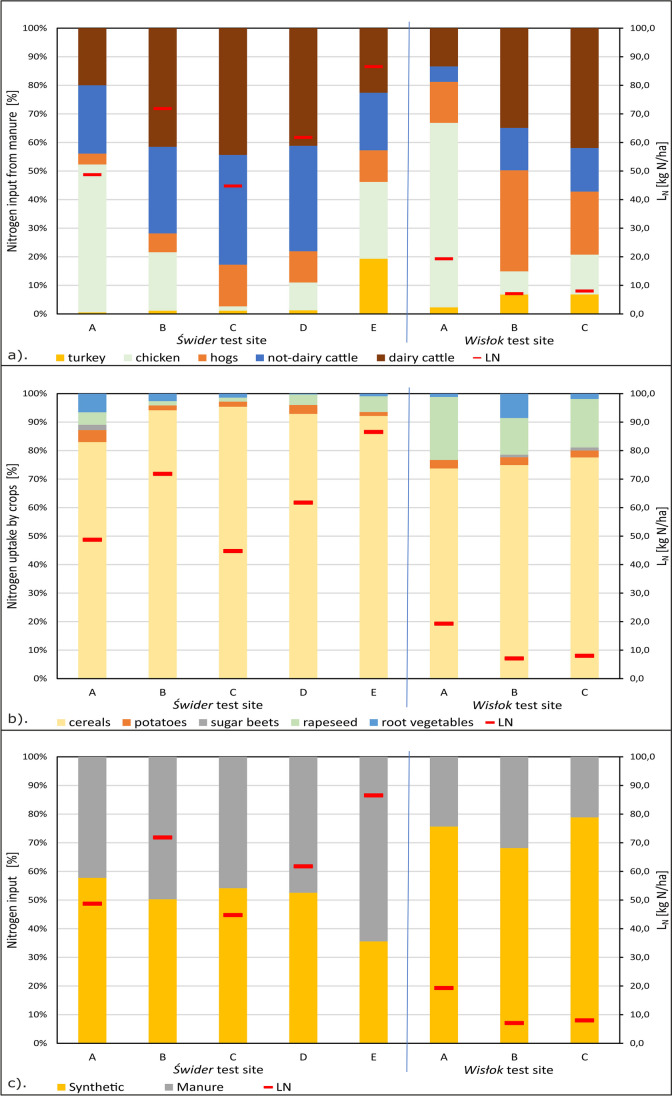
Share of (**a**) type of animal species in total nitrogen input from manure, (**b**) type of crop species in total nitrogen uptake by crops, and (**c**) type of fertilizer in total nitrogen input from fertilizing for scenario I, and result of nitrogen balance per 1 ha crops (L_N_). A, B, C, D, E—subareas in the test site

At the Świder test site, the share of individual animal species in nitrogen input from manure varies spatially depending on the subarea (Table S5 and Fig. 6a). In each of the subareas, the influence of dairy cattle and not-dairy cattle breeding is dominant—in total, it ranges from 43 to 82%. In the Wisłok test site, the share of individual species is more diverse. The share of both types of cattle is smaller, i.e., from 18 to 57%, and the share of other animal species in nitrogen input increases. It should be emphasized that the share of individual species in the total nitrogen input from manure depends directly on the number of animals of given species in subareas (Table S5).

In both test sites, the share of cereals in nitrogen uptake by crops clearly dominates (Table S5 and Fig. 6b). In the Świder test site, nitrogen uptake in four from five sub areas exceeds 90%, and that in the Wisłok test site is about 75%. The percentage share of individual crop species in the total nitrogen uptake by crops depends directly on the area under cultivation of individual species in subareas (Table S5).

At the Świder test site, the share of fertilization types (synthetic and manure) in the total nitrogen input from fertilization is generally similar in individual subareas (Table S5 and Fig. 6c). At the Wisłok test site, the share of synthetic fertilization clearly dominates—over 70%. The share of these types of fertilization depends on the number of farm animals and the area of crop cultivation in the subareas (Table S5).

Additionally, the results of the nitrogen load balance (*L*_*N*_) calculated for the subareas according to Eq. (3) are marked on Fig. 6 for scenario I (Table S5) to show their spatial differentiation. The value of the load balance *L*_*N*_ depends on all factors included in the calculation. These parameters include, among others, the type and number of farmed animals, the species and areas of crop, soil type, and the use of synthetic fertilizers. The consequence of the spatial differentiation of these parameters is the variation of nitrogen balance value—in the Świder test site subareas, this value is in the span of 45–87 kg N/ha, while in the Wisłok test site subareas it is much lower—in the span 7–19 kg N/ha. Such a significant difference in nitrogen balance *L*_*N*_ value between both test sites is mainly due to the differences in animal husbandry. In the Świder test site, nitrogen input from manure comes mainly from breeding of cattle—dairy and not-dairy (Fig. 6a), as a result of which the share of synthetic fertilizer in total fertilization is lower (Fig. 6c). In the Wisłok test site, nitrogen input from manure comes to a much lesser amount from cattle, and other animal species become more significant. As a result, synthetic fertilizing is more significant in the total nitrogen input—approximately 70% (Fig. 6c). Nitrate concentrations in leachate resulting from dividing the nitrogen input amount by the recharge rate from precipitation according to Eq. (6) are in Table S5.

**Fig. 7:**
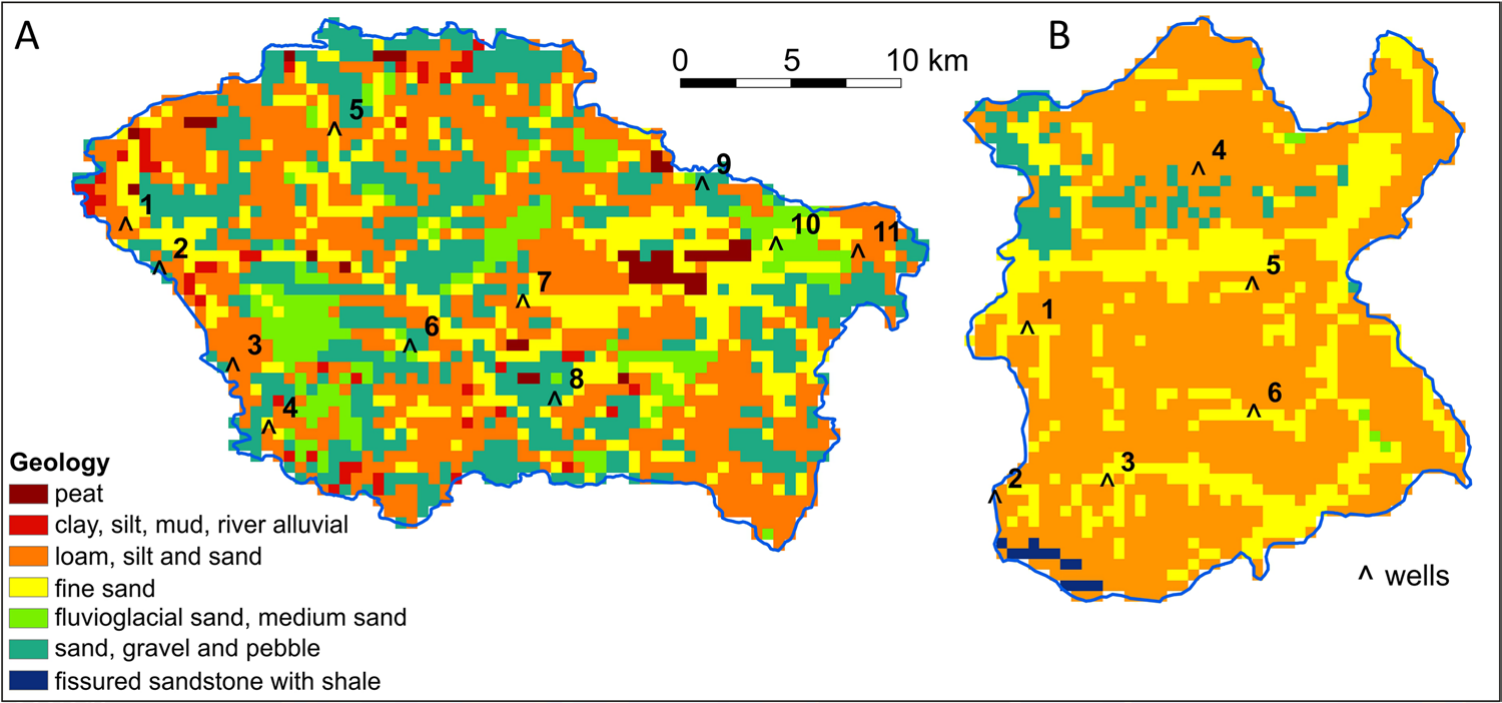
The vadose zone geology and location of the groundwater nitrate monitoring wells

Nitrate concentration in groundwater, in addition to the nitrogen load, depends on groundwater’s intrinsic vulnerability to pollution, which in turn depends on several spatially variable factors (Figs. S1 and S2). Among these factors, an important one is the geology of the vadose zone, which determines the impact of this zone on the groundwater vulnerability and pollution risk. The groundwater nitrate concentration (*NO*_*3OBS*_) acquired from the hydrogeological data base managed by the Polish Geological Institute (www.pgi.gov.pl/en) was compared with the test sites’ vadose zone geology (Table 4). The locations of the groundwater wells are presented on Fig. 7. The comparison mostly confirms the overall relationship that as the permeability of geological strata decreases, the risk of contamination and, consequently, the concentration of nitrates decreases.

**Table 4:** Comparison of nitrate concentration in groundwater (*NO*_*3OBS*_) with the test sites vadose zone geology

Świder test site			Wisłok test site		
Well no	NO_3OBS_ (mg L−1)	Geology	Well no	NO_3OBS_ (mg L−1)	Geology
1	1.8	Loam	1	17.2	Fine sand
2	40.0	Sand, gravel, and pebble	2	4.3	Loam, silt and sand
3	22.8	Loam	3	3.5	Loam, silt and sand
4	5.7	Loam/fine sand	4	2.4	Loam, silt and sand
5	4.3	Fine sand	5	4.8	Loam, silt and sand
6	2.0	Sand	6	2.7	Fine sand
7	4.4	Loam			
8	1.6	Sand, gravel, and pebble			
9	3.3	Sand, gravel, and pebble			
10	15.5	Sand			
11	80.1	Loam

The groundwater nitrate pollution risk in both test catchments affects also some groundwater-dependent ecosystems—springs, streams, and rivers. These are habitats for invertebrates, fish, and amphibians, whose health status and well-being depend on water quality.

The adoption of three scenarios with varying levels of fertilization made it possible to evaluate the sensitivity and effectiveness of the method. The spatial variations and changes in the results of the groundwater nitrate pollution risk assessment among the scenarios discussed indicate that the method is highly sensitive to changes in the values of the parameters considered in the assessment.

**Fig. 8:**
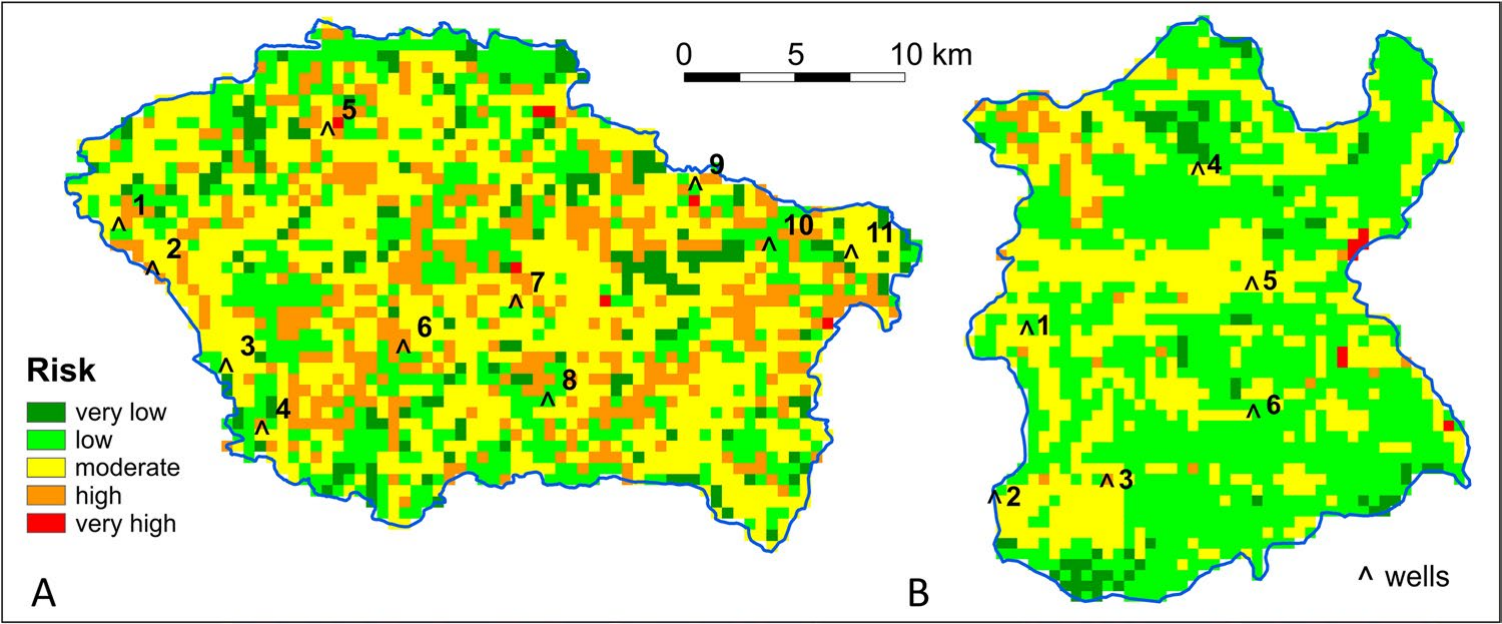
The groundwater nitrate pollution risk assessment for Scenario I and location of the monitoring wells used for assessment validation: *Świder* test site (**A**), *Wisłok* test site (**B**)

The groundwater nitrate pollution risk is not a directly measurable quantitative parameter. Risk describes the possibility of an event occurring. Therefore, the resulting nitrate pollution risk assessment, i.e., our model, was validated indirectly with the groundwater nitrate concentration (*NO*_*3OBS*_). The locations of the groundwater wells are presented on Fig. 8. Nitrate baseline, i.e., natural, concentration in the groundwater which usually does not exceed 9 mg NO_3 _L^−1^ (Shand and Edmunds 2008; Huang et al. 2013) was used as reference level for NO_3_ concentrations observed in randomly located wells. This comparison shows that NO_3_ concentrations are generally consistent with the groundwater pollution risk level assessed for scenario I in the areas where the wells are located (Table 5). Several features can contribute to minor non-compliances, including those adopted in the assessment of groundwater intrinsic vulnerability to pollution and land use—the other factors in the groundwater nitrate pollution risk assessment. The observed NO_3_ concentrations in groundwater result from the unknown level of synthetic fertilization and breeding that occurred in the past, yet our model was based on the data regarding fertilizing and breeding from 2020. In addition, nitrogen input balance omitted its losses associated with possible surface runoff and volatilization; hence, the risk obtained in our model may be slightly overestimated.

**Table 5:** Comparison of nitrate concentration in groundwater (*NO*_*3OBS*_) with nitrate pollution risk obtained for scenario I

Świder test site				Wisłok test site			
Well no	NO_3OBS_ (mg L^−1^)	Risk	Validation	Well no	NO_3OBS_ (mg L^−1^)	Risk	Validation
1	1.8	Low	** + **	1	17.2	Low	** − **
2	40.0	High	** + **	2	4.3	Moderate	** ± **
3	22.8	Moderate	** + **	3	3.5	Moderate	** ± **
4	5.7	Moderate	** ± **	4	2.4	Moderate	** ± **
5	4.3	Moderate	** ± **	5	4.8	Moderate	** ± **
6	2.0	Low	** + **	6	2.7	Low	** + **
7	4.4	Low	** + **				
8	1.6	Low	** + **				
9	3.3	Low	** + **				
10	15.5	Low	** − **				
11	80.1	Moderate	** ± **				

The changes concerned the level of synthetic and organic fertilization, which is an important factor in the risk to natural groundwater quality. An important element of this method is the balance of nitrogen loading, considering the doses of synthetic fertilizer and manure. This method enables effective management of the intensity of organic fertilization by controlling the size and nature of farms, and synthetic fertilizer application rates at the scale of an administrative unit or catchment area.

The obtained high spatial variability of groundwater nitrate pollution risk indicates that the adopted size of the individual raster 500 × 500 m proved to be optimal in the groundwater risk analysis for areas of several hundred square kilometers.

The projected reduction in groundwater nitrate pollution risk as a result of the adopted reduction in fertilization by 2030 will not reduce nitrate concentrations in aquifers to natural groundwater quality levels by that time. The reason is that the return to this level is postponed by the lag time, which may be several years (Meals et al. 2010; McDowell et al. 2021; Kaandorp et al. 2021). This phenomenon is related to the timing of the vertical transport of nitrate in the leachate to the aquifer through the vadose zone and then to the timing of the lateral transport of nitrate in the aquifer to surface water bodies.

An advantage, yet also a limitation, of this method is the consideration of the balance of nitrogen loads to quantify the impact of fertilization. This topic requires the collection of different statistics for administrative units or catchments. This task is expensive and requires organizational capacity, which may be difficult in some regions. In the absence of these data, or parts of them, the rating for the qualitative assessment of the potential adverse impact of fertilized areas presented in Table 1 can be used to approximate groundwater nitrate pollution risk preliminarily. However, without the quantitative approach adopted in this study, effective management of natural groundwater quality may be ineffective. This concern is especially true regarding the need for objective scientific support in making difficult decisions in the near future, covering an area in the triangle of topics: (i) mitigation of climate change; (ii) preservation of good groundwater status or returning to this status; and (iii) preservation of the income of the population living in high-production agriculture, including livestock husbandry.

## Conclusions

In the test area with intensive fertilization—the Świder test site—in scenario I the current total nitrogen input was 170–225 kg N ha^−1^, including 130–180 kg N ha^−1^ from fertilization, depending on the subarea. Assuming in scenario II synthetic fertilization reduced by 25% did not significantly change the groundwater nitrate pollution risk. In scenario III, the additional 50% reduction in husbandry significantly reduced the impact of fertilization and as a consequence, low-risk zones dominated. In a test area with moderate fertilization and husbandry—the Wisłok test site—similar patterns were observed, but on a smaller scale than that of the test site with intensive fertilization. In the Wisłok test site in scenario I the current total nitrogen input was 135–165 kg N ha^−1^, including 95–120 kg N ha^−1^ from fertilization, depending on the subarea. The scenario II forecast showed a reduction in risk to low and very low. This result confirmed that greater reductions in synthetic fertilization than those adopted in this scenario were unnecessary.

Predictive simulations of the groundwater nitrate pollution risk for the adopted test sites confirmed that reducing synthetic and organic fertilization had an effect, especially in areas with intensive fertilization. According to this modeling, under typical agricultural, climatic, soil, and geological conditions of Europe for the current total fertilization of 100–120 kg N ha^−1^, a reduction of synthetic nitrogen fertilization by approximately 10% can result in a low risk of degradation of natural groundwater quality. With more intense fertilization, on the order of 150–180 kg N ha^−1^, a significant reduction in total fertilization (synthetic and manure) of approximately 40–50% may be required to achieve this goal.

The novelty of the proposed methodology is a holistic approach to the issue of groundwater nitrate pollution risk assessment, based on the potential impact of land use, nitrogen balance, and vulnerability. The method allows to predict the effects of various scenarios related to levels of natural and/or synthetic fertilization. The possibility of using detailed statistical data related to agriculture increases the reliability of predictions. Future studies should focus on the analysis of the effectiveness of this method in other agricultural, soil, and geological conditions than those adopted in this assessment. The method we have adopted may help decision-makers introduce solutions to manage groundwater nitrate pollution risk. These decisions should be spatially and quantitatively differentiated, depending on total input of synthetic fertilization and manure from livestock farming.

### Supplementary Information

Below is the link to the electronic supplementary material.

Supplementary file1 (DOCX 1105 KB)

Supplementary file2 (PDF 160 KB)

Supplementary file3 (PDF 160 KB)

Supplementary file4 (PDF 161 KB)

## Data Availability

The datasets used and analyzed during the current study are available from the authors on request.
