# Supplementary material for: Groundwater nitrate pollution risk assessment based on the potential impact of land use, nitrogen balance, and vulnerability
Source: Environ Sci Pollut Res Int. 2023 Nov 16;30(58):122508–23. doi: 10.1007/s11356-023-30850-9 (PMC10724313; doi:10.1007/s11356-023-30850-9)
Supplement: Supplementary file 1 — Supplementary file1 (DOCX 1105 KB) [file 11356_2023_30850_MOESM1_ESM.docx]

**Environmental Science and Pollution Research**

**Groundwater nitrate pollution risk assessment based on the potential impact of land use, nitrogen balance and vulnerability**

Robert Duda^1*^, Robert Zdechlik^1^, Jarosław Kania^1^

*^1^AGH University of Science and Technology; Faculty of Geology, Geophysics and Environmental Protection, Mickiewicza 30, 30-059 Kraków, Poland.*

*duda@agh.edu.pl

**Supplementary Material**

**Table S1**

Average mineral nitrogen stock in topsoil (*N_s_*) in spring, depending on soil category (based on Jadczyszyn, 2006; Jadczyszyn et al., 2012; CM, 2020) (after Duda et al., 2021)

| Soil  category | *N_s_*  (kg⋅ha^-1^) | Soil type |
| --- | --- | --- |
| very light | 49 | sand, sandy soil, rocky soil, skeletal soil |
| light | 59 | silty sand, sandy silt, rendzina, sandy alluvial soil, muck soil |
| medium | 62 | sandy loam, loamy silt, slightly loamy soil, silt soil, loess, alluvial soil |
| heavy | 66 | silty loam, clayey silt, silty clay, loamy soil, clay soil, muck bog soil |

**Table S2**

Soil nitrogen (*α*) and manure nitrogen from European livestock breeds (*β*) efficiency (based on CM, 2020; Klages et al., 2020, averaged)

| Nitrogen source | Factor | |
| --- | --- | --- |
| mineral nitrogen from soil | *α* | 0.75 |
| slurry and solid manure  (cattle, hogs, poultry) | *β* | 0.45 |

**Table S3**

Approximate rates of nitrogen contained in manure by livestock categories (based on Olecka et al., 2019, averaged)

| Animal species  (European breeds) | kgN⋅head^-1^⋅year^-1^ |
| --- | --- |
| dairy cattle | 75 |
| not-dairy cattle | 45 |
| hogs | 15 |
| turkey | 1.5 |
| chicken | 0.5 |

**Table S4**

Nitrogen amount (*N_d_*) which a crop can uptake from medium soil^1)2)^ (based on CM, 2020, averaged)

| Crop | Nitrogen  (kg⋅ha^-1^⋅y^-1^) |  | Crop | Nitrogen  (kg⋅ha^-1^⋅y^-1^) |
| --- | --- | --- | --- | --- |
| cereals (on average) | 140 |  | canola | 210 |
| potato (on average) | 135 |  | vegetables (on average) | 210 |
| sugar beets | 180 |  |  |  |

^1)^ in light topsoil (Table S1) the maximum amount of nitrogen from all sources is reduced by about 10%, in very light soil by 20%, and in heavy soil it is increased by 10%,

^2)^ the nitrogen amount stated in the table is the sum of approximate nitrogen amounts originated from all sources (synthetic fertilizer, manure, and soil nitrogen) determined for crop optimal yields under temperate climate conditions and soils typical in Europe as well as balanced application of nitrogen, phosphate and potassium (N-P-K) fertilizers, and pesticides.

**Table S5**

Nitrogen balance and nitrate concentration in leachate calculation, and potential adverse impact of fertilization assessment in test areas for adopted scenarios. Scenario I, Scenario II, Scenario III

(*in separate files*)

**References**

CM *Council of Ministers,* 2020. The action program aimed at reducing water pollution by nitrates from agricultural sources and to prevent further pollution. Regulation of the Council of Ministers of the Republic of Poland, Dziennik Ustaw 2020, 243 (in Polish)

Duda, R., Zdechlik, R., Kania, J., 2021. Semiquantitative risk assessment method for groundwater source protection using a process-based interdisciplinary approach. Water Resour. Manag. 35, 3373–3394. https://doi.org/10.1007/s11269-021-02898-0

Jadczyszyn, T., 2006. Selected aspects of agrochemical soil testing. In: Studia i Raporty, Institute of Soil Science and Plant Cultivation (IUNG), Puławy (in Polish)

Jadczyszyn, T., Kowalczyk, J., Lipiński, W., 2012. Mineral fertilization on arable land and permanent grassland. Instrukcja Rozpowszechniania 184:1–24, Institute of Soil Science and Plant Cultivation (IUNG), Puławy (in Polish)

Klages, S., Heidecke, C., Osterburg, B., Bailey, J., Calciu, I. et al. 2020. Nitrogen surplus − A uniﬁed indicator for water pollution in Europe? Water 12, 1197. https://doi.org/10.3390/w12041197.

Olecka, A., Bebkiewicz, K., Chłopek, Z., Dębski, B., Doberska, A. et al. 2019. Poland’s National Inventory Report 2019. Greenhouse Gas Inventory for 1988-2017. Submission under the UN Framework Convention on Climate Change and its Kyoto Protocol. National Centre for Emission Management (KOBiZE) at the Institute of Environmental Protection – National Research Institute, Warsaw. https://www.kobize.pl/uploads/materialy/materialy_do_pobrania/krajowa_inwentaryzacja_emisji/NIR_POL_2019_23.05.2019.pdf


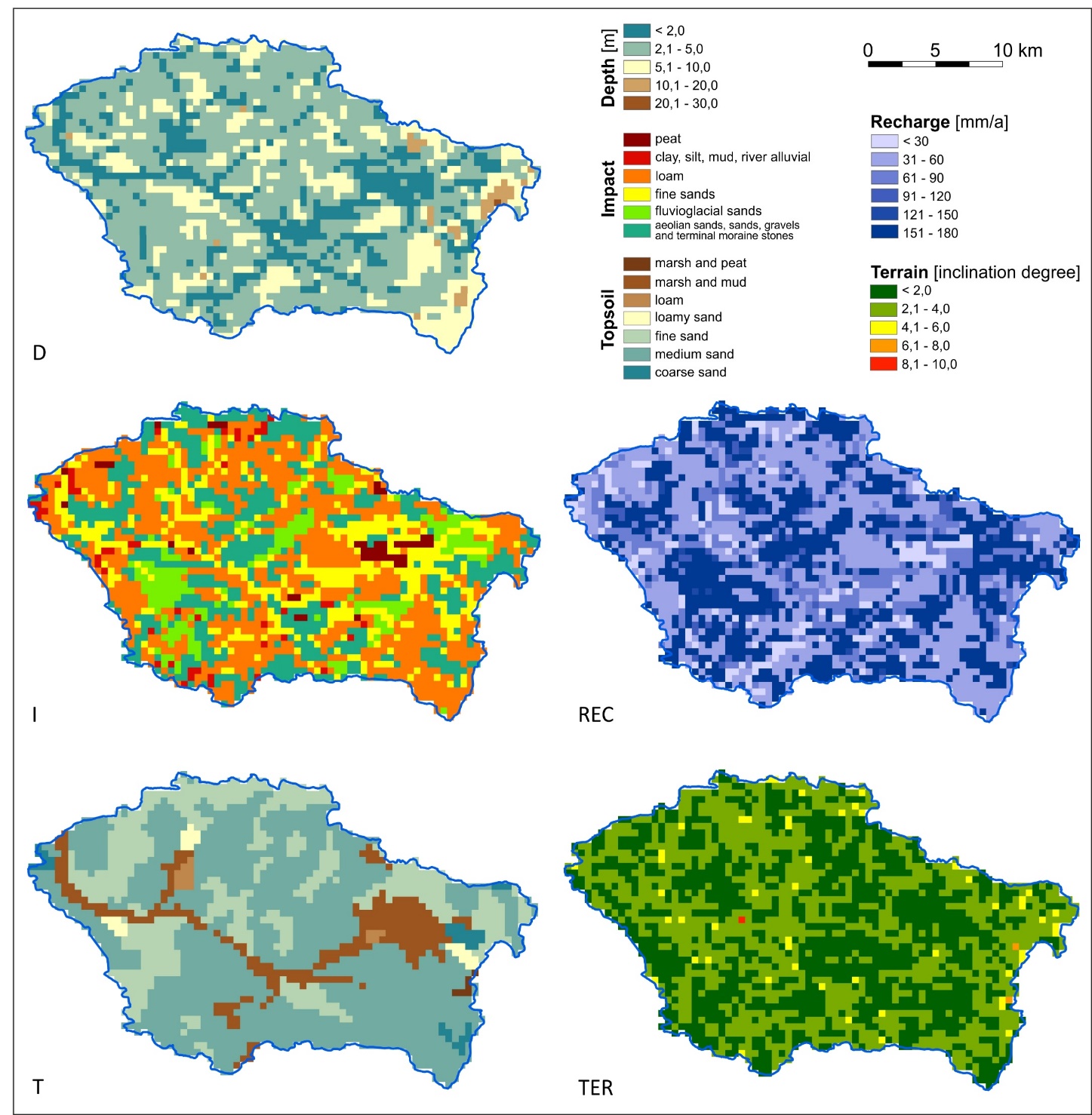


**Fig. S1** Maps of the depth to the groundwater table (D), impact of the lithology of the vadose zone (I), net recharge (REC), topsoil type (T), and terrain topography (TER) – *Świder* test site


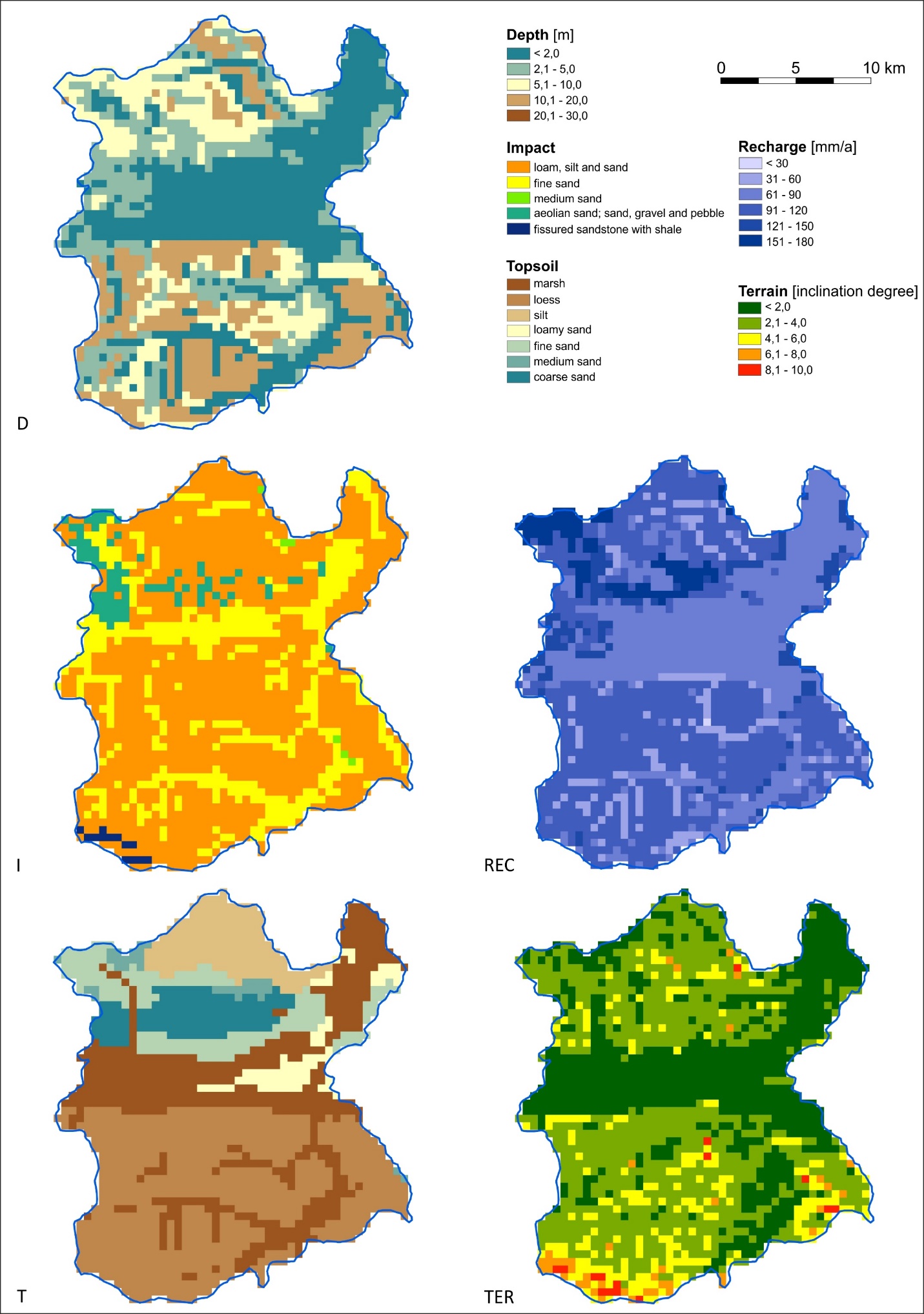


**Fig. S2** Maps of the depth to the groundwater table (D), impact of the lithology of the vadose zone (I), net recharge (REC), topsoil type (T), and terrain topography (TER) – *Wisłok* test site
