# Supplementary material for: Groundwater nitrate pollution risk assessment based on the potential impact of land use, nitrogen balance, and vulnerability
Source: Environ Sci Pollut Res Int. 2023 Nov 16;30(58):122508–23. doi: 10.1007/s11356-023-30850-9 (PMC10724313; doi:10.1007/s11356-023-30850-9)
Supplement: Supplementary file 2 — Supplementary file2 (PDF 160 KB) [file 11356_2023_30850_MOESM2_ESM.pdf]

Table S5. Nitrogen balance and nitrate concentration in leachate calculation, and potential adverse impact of fertilization assessment in test areas for adopted scenarios. Scenario I

Test site		Świder						Wisłok			
Administrative unit		**A**	**B**	**C**	**D**	**E**	**Total**	**A**	**B**	**C**	**Total**
Area	ha	6 203	28 726	14 567	1 474	4 195	55 166	16 480	8 048	19 704	44 232
CROPS											
Crops area	**ha**	***665***	***5 553***	***3 605***	***513***	***1 251***	*11 586*	***4 339***	***1 792***	***6 295***	*12 425*
cereals	ha	*574*	*5 281*	*3 469*	*482*	*1 178*		*3 464*	*1 447*	*5 222*	
potatoes	ha	*30*	*99*	*68*	*17*	*18*		*146*	*55*	*169*	
sugar beets	ha	*10*	*0*	*0*	*0*	*0*		*0*	*14*	*56*	
rapeseed	ha	*20*	*74*	*34*	*13*	*47*		*693*	*165*	*762*	
root vegetables	ha	*30*	*99*	*34*	*1*	*8*		*36*	*110*	*85*	
NITROGEN INPUT FROM SYNTHETIC FERTILIZER											
**N_{am} - synthetic fertilizer input**	**kg N**	**70 493**	**616 976**	**362 748**	**57 107**	**117 775**	**1 225 099**	**441 176**	**151 589**	**621 036**	**1 213 801**
**N_{am_1ha} - synthetic fertilizer input per 1 ha crops**	**kg N/ha**	**106**	**111**	**101**	**111**	**94**	**106**	**102**	**85**	**99**	**98**
SOIL NITROGEN											
very light soil [1)]	ha	*0*	*5610*	*390*	*20*	*2720*		*6400*	*3580*	*610*	
light soil	ha	*4380*	*8160*	*1670*	*0*	*0*		*0*	*0*	*0*	
medium soil	ha	*900*	*11660*	*10780*	*1460*	*1470*		*10080*	*4470*	*19090*	
heavy soil	ha	*920*	*3290*	*1720*	*0*	*0*		*0*	*0*	*0*	
**EFFECTIVE SOIL NITROGEN**											
N_s - soil nitrogen stock [2)]	kg N	374 940	1 696 390	899 520	91 500	224 420	3 286 770	938 560	452 560	1 213 470	2 604 590
soil nitrogen effectiveness relative to synthetic fertilizer nitrogen [3)]		0.75	0.75	0.75	0.75	0.75	0.75	0.75	0.75	0.75	0.75
N_{as} - effective soil nitrogen input	kg N	281 205	1 272 293	674 640	68 625	168 315	2 465 078	703 920	339 420	910 103	1 953 443
**N_{as} - effective soil nitrogen input on crops area**	**kg N**	**30 133**	**245 992**	**166 976**	**23 872**	**50 175**	**517 148**	**185 390**	**75 593**	**290 755**	**551 738**
**N_{as_1ha} - effective soil nitrogen input per 1 ha crops**	kg N/ha	**45**	**44**	**46**	**47**	**40**	**45**	**43**	**42**	**46**	**44**
NITROGEN FROM MANURE INPUT											
dairy cattle		*137*	*3 378*	*1 817*	*283*	*643*		*253*	*329*	*931*	
not-dairy cattle		*274*	*4 107*	*2 621*	*423*	*955*		*170*	*234*	*564*	
hogs		*131*	*2 681*	*2 988*	*375*	*1 574*		*1 357*	*1 669*	*2 448*	
chicken		*53 355*	*249 607*	*9 216*	*10 050*	*114 580*		*183 316*	*11 469*	*46 370*	
turkey		*188*	*4 617*	*2 341*	*432*	*27 489*		*2 180*	*3 207*	*7 560*	
**nitrogen input from manure** [4)]											
dairy cattle	kg N	10 302	253 336	136 269	21 222	48 229		19 005	24 712	69 814	
not-dairy cattle	kg N	12 326	184 830	117 928	19 023	42 972		7 662	10 511	25 374	
hogs	kg N	1 958	40 213	44 817	5 629	23 613		20 362	25 041	36 722	
chicken	kg N	26 678	124 803	4 608	5 025	57 290		91 658	5 734	23 185	
turkey	kg N	282	6 926	3 511	647	41 233		3 270	4 811	11 340	
N_n - total nitrogen input from manure on crops area	**kg N**	**51 546**	**610 109**	**307 133**	**51 546**	**213 337**	**1 233 671**	**141 957**	**70 810**	**166 435**	**379 202**
**EFFECTIVE NITROGEN FROM MANURE** [5)]											
dairy cattle	kg N	4 636	114 001	61 321	9 550	21 703		8 552	11 121	31 416	
not-dairy cattle	kg N	5 547	83 173	53 068	8 560	19 338		3 448	4 730	11 418	
hogs	kg N	881	18 096	20 168	2 533	10 626		9 163	11 269	16 525	
chicken	kg N	12 005	56 162	2 074	2 261	25 780		41 246	2 580	10 433	
turkey	kg N	127	3 117	1 580	291	18 555		1 471	2 165	5 103	
**N_{an} - effective nitrogen input from manure on crops area**	**kg N**	**23 196**	**274 549**	**138 210**	**23 196**	**96 002**	**555 152**	**63 881**	**31 865**	**74 896**	**170 641**
**N_{an_1ha} - effective nitrogen input from manure per 1 ha crops**	**kg N/ha**	**35**	**49**	**38**	**45**	**77**	**48**	**15**	**18**	**12**	**14**
NITROGEN UPTAKE BY CROPS [6)]											
very light soil											
cereals	kg N	0	115 542	10 406	730	85 630		150 658	72 073	18 111	
potatoes	kg N	0	2 083	197	25	1 284		6 117	2 648	566	
sugar beets	kg N	0	0	0	0	0		0	883	252	
rapeseed	kg N	0	2 430	153	29	5 138		45 197	12 355	3 965	
root vegetables	kg N	0	3 239	153	2	856		2 379	8 237	441	
light soil											
cereals	kg N	51 095	189 068	50 131	0	0		0	0	0	
potatoes	kg N	2 593	3 408	948	0	0		0	0	0	
sugar beets	kg N	1 153	0	0	0	0		0	0	0	
rapeseed	kg N	2 689	3 976	737	0	0		0	0	0	
root vegetables	kg N	4 034	5 301	737	0	0		0	0	0	
medium soil											
cereals	kg N	11 665	300 182	359 552	66 601	57 847		296 607	112 487	708 491	
potatoes	kg N	592	5 410	6 798	2 253	868		12 043	4 132	22 157	
sugar beets	kg N	263	0	0	0	0		0	1 377	9 848	
rapeseed	kg N	614		5 288	2 629	3 471		88 982	19 284	155 102	
root vegetables	kg N	921	8 416	5 288	219	578		4 683	12 856	17 234	
heavy soil											
cereals	kg N	13 117	93 170	63 105	0	0		0	0	0	
potatoes	kg N	666	1 679	1 193	0	0		0	0	0	
sugar beets	kg N	296	0	0	0	0		0	0	0	
rapeseed	kg N	690	1 959	928	0	0		0	0	0	
root vegetables	kg N	1 036	2 612	928	0	0		0	0	0	
**N_d - nitrogen uptake by crops**	**kg N**	**91 424**	**738 475**	**506 542**	**72 488**	**155 673**	**1 564 601**	**606 666**	**246 331**	**936 166**	**1 789 163**
F_N - ADVERSE IMPACT OF FERTILIZATION											
L_N - result of nitrogen balance on crops area [7)]	kg N	32 398	399 043	161 392	31 687	108 279		83 780	12 716	50 520	
L_N - result of nitrogen balance per 1 ha crops	kg N/ha	48.7	71.9	44.8	61.8	86.6		19.3	7.1	8.0	
RCH - aquifer recharge	mm/year	95	112	113	101	104		98	94	86	
**C_{NO3} - nitrate concentration in leachate**	**$mgNO_3/L$**	**227**	**284**	**176**	**271**	**369**		**87**	**33**	**41**	
ADVERSE IMPACT OF FERTILIZATION - RATING											
**F_N**		**4**	**4**	**4**	**4**	**4**		**3**	**2**	**2**	

[(1)] soil category according to Table S1; [(2)] according to Table S1; [(3)] according to Table S2; [(4)] according to Table S3, [(5)] according to Table S2, [(6)] according to Table S4,
[(7)] a positive value means surplus
