# Supplementary material for: Groundwater nitrate pollution risk assessment based on the potential impact of land use, nitrogen balance, and vulnerability
Source: Environ Sci Pollut Res Int. 2023 Nov 16;30(58):122508–23. doi: 10.1007/s11356-023-30850-9 (PMC10724313; doi:10.1007/s11356-023-30850-9)
Supplement: Supplementary file 4 — Supplementary file4 (PDF 161 KB) [file 11356_2023_30850_MOESM4_ESM.pdf]

Table S5. Nitrogen balance and nitrate concentration in leachate calculation, and potential adverse impact of fertilization assessment in test areas for adopted scenarios. Scenario III

Test site		Świder						Wisłok			
Administrative unit		A	B	C	D	E	Total	A	B	C	Total
Area	ha	6 203	28 726	14 567	1 474	4 195	55 166	16 480	8 048	19 704	44 232
CROPS											
Crops area	**ha**	***665***	***5 553***	***3 605***	***513***	***1 251***	*11 586*	***4 339***	***1 792***	***6 295***	*12 425*
cereals	ha	*574*	*5 281*	*3 469*	*482*	*1 178*		*3 464*	*1 447*	*5 222*	
potatoes	ha	*30*	*99*	*68*	*17*	*18*		*146*	*55*	*169*	
sugar beets	ha	*10*	*0*	*0*	*0*	*0*		*0*	*14*	*56*	
rapeseed	ha	*20*	*74*	*34*	*13*	*47*		*693*	*165*	*762*	
root vegetables	ha	*30*	*99*	*34*	*1*	*8*		*36*	*110*	*85*	
NITROGEN INPUT FROM SYNTHETIC FERTILIZER											
**N_{am} - synthetic fertilizer input**	**kg N**	**52 870**	**462 732**	**272 061**	**42 830**	**88 331**	**918 824**	**397 058**	**136 430**	**558 932**	**1 092 420**
**N_{am_1ha} - synthetic fertilizer input per 1 ha crops**	**kg N/ha**	**80**	**83**	**75**	**84**	**71**	**79**	**92**	**76**	**89**	**88**
SOIL NITROGEN											
very light soil [1]	ha	*0*	*5610*	*390*	*20*	*2720*		*6400*	*3580*	*610*	
light soil	ha	*4380*	*8160*	*1670*	*0*	*0*		*0*	*0*	*0*	
medium soil	ha	*900*	*11660*	*10780*	*1460*	*1470*		*10080*	*4470*	*19090*	
heavy soil	ha	*920*	*3290*	*1720*	*0*	*0*		*0*	*0*	*0*	
**EFFECTIVE SOIL NITROGEN**											
N_s - soil nitrogen stock [2]	kg N	374 940	1 696 390	899 520	91 500	224 420	3 286 770	938 560	452 560	1 213 470	2 604 590
soil nitrogen effectiveness relative to synthetic fertilizer nitrogen [3]		0.75	0.75	0.75	0.75	0.75	0.75	0.75	0.75	0.75	0.75
N_{as} - effective soil nitrogen input	kg N	281 205	1 272 293	674 640	68 625	168 315	2 465 078	703 920	339 420	910 103	1 953 443
**N_{as} - effective soil nitrogen input on crops area**	**kg N**	**30 133**	**245 992**	**166 976**	**23 872**	**50 175**	**517 148**	**185 390**	**75 593**	**290 755**	**551 738**
**N_{as_1ha} - effective soil nitrogen input per 1 ha crops**	kg N/ha	**45**	**44**	**46**	**47**	**40**	**45**	**43**	**42**	**46**	**44**
NITROGEN FROM MANURE INPUT											
dairy cattle		*69*	*1 689*	*908*	*141*	*322*		*253*	*329*	*931*	
not-dairy cattle		*137*	*2 054*	*1 310*	*211*	*477*		*170*	*234*	*564*	
hogs		*65*	*1 340*	*1 494*	*188*	*787*		*1 357*	*1 669*	*2 448*	
chicken		*26 678*	*124 803*	*4 608*	*5 025*	*57 290*		*183 316*	*11 469*	*46 370*	
turkey		*94*	*2 309*	*1 170*	*216*	*13 744*		*2 180*	*3 207*	*7 560*	
**nitrogen input from manure** [4]											
dairy cattle	kg N	5 151	126 668	68 134	10 611	24 114		19 005	24 712	69 814	
not-dairy cattle	kg N	6 163	92 415	58 964	9 511	21 486		7 662	10 511	25 374	
hogs	kg N	979	20 107	22 408	2 814	11 806		20 362	25 041	36 722	
chicken	kg N	13 339	62 402	2 304	2 513	28 645		91 658	5 734	23 185	
turkey	kg N	141	3 463	1 756	324	20 617		3 270	4 811	11 340	
N_n - total nitrogen input from manure on crops area	**kg N**	**25 773**	**305 055**	**153 566**	**25 773**	**106 669**	**616 836**	**141 957**	**70 810**	**166 435**	**379 202**
**EFFECTIVE NITROGEN FROM MANURE** [5]											
dairy cattle	kg N	2 318	57 001	30 660	4 775	10 851		8 552	11 121	31 416	
not-dairy cattle	kg N	2 773	41 587	26 534	4 280	9 669		3 448	4 730	11 418	
hogs	kg N	440	9 048	10 084	1 266	5 313		9 163	11 269	16 525	
chicken	kg N	6 002	28 081	1 037	1 131	12 890		41 246	2 580	10 433	
turkey	kg N	63	1 558	790	146	9 277		1 471	2 165	5 103	
**N_{an} - effective nitrogen input from manure on crops area**	**kg N**	**11 598**	**137 275**	**69 105**	**11 598**	**48 001**	**277 576**	**63 881**	**31 865**	**74 896**	**170 641**
**N_{an_1ha} - effective nitrogen input from manure per 1 ha crops**	**kg N/ha**	**17**	**25**	**19**	**23**	**38**	**24**	**15**	**18**	**12**	**14**
NITROGEN UPTAKE BY CROPS [6]											
very light soil											
cereals	kg N	0	115 542	10 406	730	85 630		150 658	72 073	18 111	
potatoes	kg N	0	2 083	197	25	1 284		6 117	2 648	566	
sugar beets	kg N	0	0	0	0	0		0	883	252	
rapeseed	kg N	0	2 430	153	29	5 138		45 197	12 355	3 965	
root vegetables	kg N	0	3 239	153	2	856		2 379	8 237	441	
light soil											
cereals	kg N	51 095	189 068	50 131	0	0		0	0	0	
potatoes	kg N	2 593	3 408	948	0	0		0	0	0	
sugar beets	kg N	1 153	0	0	0	0		0	0	0	
rapeseed	kg N	2 689	3 976	737	0	0		0	0	0	
root vegetables	kg N	4 034	5 301	737	0	0		0	0	0	
medium soil											
cereals	kg N	11 665	300 182	359 552	66 601	57 847		296 607	112 487	708 491	
potatoes	kg N	592	5 410	6 798	2 253	868		12 043	4 132	22 157	
sugar beets	kg N	263	0	0	0	0		0	1 377	9 848	
rapeseed	kg N	614		5 288	2 629	3 471		88 982	19 284	155 102	
root vegetables	kg N	921	8 416	5 288	219	578		4 683	12 856	17 234	
heavy soil											
cereals	kg N	13 117	93 170	63 105	0	0		0	0	0	
potatoes	kg N	666	1 679	1 193	0	0		0	0	0	
sugar beets	kg N	296	0	0	0	0		0	0	0	
rapeseed	kg N	690	1 959	928	0	0		0	0	0	
root vegetables	kg N	1 036	2 612	928	0	0		0	0	0	
**N_d - nitrogen uptake by crops**	**kg N**	**91 424**	**738 475**	**506 542**	**72 488**	**155 673**	**1 564 601**	**606 666**	**246 331**	**936 166**	**1 789 163**
F_N - ADVERSE IMPACT OF FERTILIZATION											
L_N - result of nitrogen balance on crops area [7]	kg N	3 177	107 524	1 600	5 813	30 834		39 663	-2 443	-11 583	
L_N - result of nitrogen balance per 1 ha crops	kg N/ha	4.8	19.4	0.4	11.3	24.6		9.1	-1.4	-1.8	
RCH - aquifer recharge	mm/year	95	112	113	101	104		98	94	86	
**C_{NO3} - nitrate concentration in leachate**	**$mgNO_3/L$**	**22**	**77**	**2**	**50**	**105**		**41**	**0**	**0**	
ADVERSE IMPACT OF FERTILIZATION - RATING											
**F_N**		**2**	**3**	**0**	**2**	**4**		**2**	**0**	**0**	

(1) soil category according to Table S1; (2) according to Table S1; (3) according to Table S2; (4) according to Table S3, (5) according to Table S2, (6) according to Table S4,
(7) a positive value means surplus, a negative value means shortage
